# Neurosymptomatic HIV-1 CSF escape is associated with replication in CNS T cells and inflammation

**DOI:** 10.1172/JCI176358

**Published:** 2024-10-01

**Authors:** Laura P. Kincer, Ameet Dravid, Mattia Trunfio, Andrea Calcagno, Shuntai Zhou, Riccardo Vercesi, Serena Spudich, Magnus Gisslen, Richard W. Price, Paola Cinque, Sarah B. Joseph

**Affiliations:** 1 Lineberger Comprehensive Cancer Center, University of North Carolina at Chapel Hill, Chapel Hill, North Carolina, USA.; 2 Department of Medicine, Poona Hospital and Research Center, Pune, India.; 3 Ruby Hall Clinic, Pune, India.; 4 Unit of Infectious Diseases, Department of Medical Sciences, University of Turin at the “Amedeo di Savoia” Hospital, Torino, Italy.; 5 ASL “CIttà di Torino,” Torino, Italy.; 6 Unit of Infectious Diseases, San Raffaele Scientific Institute, Milan, Italy.; 7 Department of Neurology, Yale University, New Haven, Connecticut, USA.; 8 Department of Infectious Diseases, Institute of Biomedicine, Sahlgrenska Academy at the University of Gothenburg, Gothenburg, Sweden.; 9 Department of Infectious Diseases, Region Västra Götaland, Sahlgrenska University Hospital, Gothenburg, Sweden.; 10 Public Health Agency of Sweden, Solna, Sweden.; 11 Department of Neurology, University of California San Francisco, San Francisco, California, USA.; 12 Department of Microbiology and Immunology and; 13 UNC HIV Cure Center, University of North Carolina at Chapel Hill, Chapel Hill, North Carolina, USA.

**Keywords:** AIDS/HIV, Neurological disorders, T cells

## Abstract

During antiretroviral therapy (ART), most people living with HIV-1 have undetectable HIV-1 RNA in their plasma. However, they occasionally present with new or progressive neurologic deficits and detectable HIV-1 RNA in the cerebrospinal fluid (CSF), a condition defined as neurosymptomatic HIV-1 CSF escape (NSE). We explored the source of neuropathogenesis and HIV-1 RNA in the CSF during NSE by characterizing HIV-1 populations and inflammatory biomarkers in CSF from 25 individuals with NSE. HIV-1 populations in the CSF were uniformly drug resistant and adapted to replication in CD4^+^ T cells, but differed greatly in genetic diversity, with some having low levels of diversity similar to those observed during untreated primary infection and others having high levels like those during untreated chronic infection. Higher diversity in the CSF during NSE was associated with greater CNS inflammation. Finally, optimization of ART regimen was associated with viral suppression and improvement of neurologic symptoms. These results are consistent with CNS inflammation and neurologic injury during NSE being driven by replication of partially drug-resistant virus in CNS CD4^+^ T cells. This is unlike nonsuppressible viremia in the plasma during ART, which typically lacks clinical consequences and is generated by virus expression without replication.

## Abstract 2

People on antiretroviral therapy with undetectable HIV-1 in their blood can experience severe neurologic symptoms due to virus replication in T cells in their CNS.

## Graphical abstract

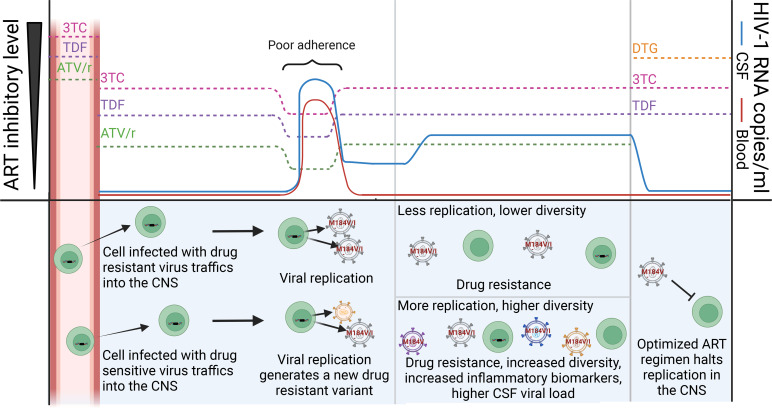

## Introduction

The CNS is an immune-privileged compartment where inflammation is typically limited due to its potential pathogenic effects. For example, neuroinflammation is known to be a contributor in the development of neurodegenerative diseases such as Alzheimer’s disease, Parkinson’s disease, amyotrophic lateral sclerosis, and multiple sclerosis (1). In addition, neuroinflammation in response to viral, bacterial, or fungal pathogens can generate severe neuropathogenesis and encephalitis (2).

During untreated infection, HIV-1 replication in CD4^+^ T lymphocytes and myeloid cells (macrophages and microglia) in the CNS is associated with inflammation (3) and severe neurologic and neurocognitive complications (4, 5). Myeloid lineage cells are prevalent in the CNS, and HIV-1 infection of myeloid cells in the CNS is a central feature of HIV encephalitis in late-stage untreated infection manifesting as HIV-associated dementia (HAD) (4, 5). In contrast, CD4^+^ T cells are typically rare in the brain (6, 7), though it is now appreciated that they are always present in the CNS (6), including at elevated levels during early HIV-1 infection (8, 9). This often corresponds with a rise in CD8^+^ T cells within the CNS (10–13).

The implementation of antiretroviral therapy (ART) has greatly reduced rates of severe neurocognitive impairment (14–16) and typically suppresses viral RNA to undetectable levels in both the periphery and CNS (17). Despite these improvements, mild neurocognitive impairment and depression remain common in people living with HIV (PLWH) (14–16), and HIV-1 RNA can occasionally be detected in cerebrospinal fluid (CSF) (18, 19) or brain tissue (20, 21) collected from people on ART. There are many mechanisms that may explain persistent neuropathogenesis during ART, including damage that took place prior to ART, continued virus replication and neuroinflammation, toxicity of ART, coinfections, lifestyle causes, or other underlying factors (reviewed in ref. 22). Studies have, however, observed that neuropathogenesis during ART is correlated with the presence of HIV-1 in the CNS (23). For example, detection of HIV-infected cells in the CSF is associated with poorer performance on neuropsychological tests (24), and HIV-1 cell–associated RNA transcripts in CSF cells are associated with brain injury (19). Therefore, there is a great interest in understanding the source of virus in the CNS during ART and whether it contributes to neuropathogenesis.

Residual viral RNA in the CNS during ART can either be due to ongoing virus replication or virus expression without replication. Replication can occur if the virus is drug resistant or if drug levels in the CNS are noninhibitory. However, ART intensification studies suggest that the latter is not a major driver of detectable levels of CSF HIV-1 RNA in most people on ART (25, 26). Alternatively, virus in the CSF could be generated by infected cells expressing HIV-1 virions in the absence of replication. Such virions could be infectious but unable to infect other cells because ART prevents entry, reverse transcription, nuclear import, and/or integration. Alternatively, virions could be noninfectious due to virion defects, including being immature if produced in the presence of protease inhibitor (PI). The possibility that virions in the CSF could be produced without replication is consistent with studies showing that HIV-1 RNA in the blood during ART is often defective (27, 28) and produced by expression, not replication (27–33).

A rare subset of PLWH and people on ART experience CSF escape in which HIV-1 RNA levels are suppressed in the blood, but remain elevated in the CSF. CSF escape can occur in the absence of neurologic symptoms (i.e., asymptomatic CSF escape [ASE], refs. 34, 35) or with severe neurologic symptoms (i.e., neurosymptomatic HIV-1 CSF escape [NSE], ref. 10–13). We previously performed a detailed genetic and phenotypic analysis of 2 individuals with ASE (18) and observed that one participant had episodic escape in which the CSF contained a homogeneous virus population likely produced by an expanding CD4^+^ T cell clone in the absence of replication. In contrast, the second participant had a persistent, drug-resistant population of macrophage-tropic virus in the CSF produced by sustained replication in macrophage/microglia during ART (18). These results suggest that ASE may emerge via multiple mechanisms.

This study was designed to identify the cellular source of NSE virus, the conditions needed for NSE, and potential mechanisms generating severe neurologic symptoms in these individuals. We addressed these questions in a rare cohort of 25 PLWH on ART who all met our definition of NSE, i.e., manifested new or worsening neurologic symptoms and signs despite well-controlled blood plasma HIV-1 RNA (i.e, less than 500 HIV-1 RNA copies/ml) and CSF HIV-1 RNA concentrations more than 0.5 logs greater than HIV-1 RNA concentrations in the plasma. We analyzed samples collected at the time of NSE diagnosis for all participants, and additional NSE time points were analyzed for a subset of participants. We used sequencing and phylogenetic analyses to assess viral diversity and determine whether NSE virus is produced by HIV-1 replication or continued virus production from CNS cells without replication and whether NSE virus was drug resistant. We also examined the cellular tropism of NSE virus in order to infer the cell type likely producing NSE virus. Finally, we used CSF biomarkers to assess the inflammatory environment during NSE and explore potential mechanisms that may generate inflammation and severe neurologic symptoms during NSE.

## Results

### NSE is observed in a subset of PLWH on otherwise suppressive ART.

We analyzed CSF from 25 study participants (Table 1) living with HIV-1 on ART who presented with a range of neurological symptoms and complaints (Supplemental Table 1; supplemental material available online with this article; https://doi.org/10.1172/JCI176358DS1). All 25 participants were diagnosed clinically with NSE using the following criteria: (a) clinical presentation with new neurologic symptoms and signs without an alternative cause, (b) taking ART with suppression of plasma HIV-1 RNA to below 500 HIV-1 RNA copies/ml, and (c) a CSF HIV-1 RNA greater than that of plasma HIV-1 RNA. Archived CSF samples were collected between 2000 and 2019 at 5 sites: San Francisco, California, USA (*n* = 7), Pune, India (*n* = 12), Milan and Turin, Italy (*n* = 3 and *n* = 1, respectively), and Gothenburg, Sweden (*n* = 2).

The main clinical features of the NSE cohort are shown in Table 1. ART regimens and drug-resistance mutations are shown in Table 2. The median CSF HIV-1 RNA was 1,700 copies/ml, and the median plasma HIV-1 RNA was 86 copies/ml. Most (92%, 23/25) participants were on a PI-based ART regimen. Atazanavir (ATV) was very common among those on a PI-containing regimen (74%, 17/23). The median and nadir blood CD4^+^ T cell counts were 427 cells/μL and 98 cells/μL, respectively. Additionally, all participants examined (24/24) had elevated CSF white blood cells (5 cells or more per μL), with a median of 14 cells/μL (Table 1).

To better understand the source of NSE virus and symptoms, we compared clinical and virologic characteristics of participants with NSE to characteristics of participants in 4 cohorts of PLWH (Supplemental Table 2). (a) The first cohort consisted of those with untreated primary HIV-1 infection (*n* = 7). Participants were from a previously described cohort of people enrolled in the US within 12 months of infection and ART naive (5). (b) The second cohort consisted of those with untreated chronic HIV-1 infection (*n* = 7). Participants were enrolled in the Tropism of HIV-1 Inflammation and NeuroCognition (THINC) study. All participants had CD4^+^ T cell counts below 400 cells/μl, were ART naive, and lacked neurologic symptoms. (c) The third cohort consisted of those who were ART-treated and virologically suppressed (*n* = 49). All participants had HIV-1 RNA levels in the blood and CSF that were undetectable by standard clinical assays and lacked neurologic symptoms. (d) The fourth cohort consisted of 2 previously characterized people with ASE (18). Both individuals were on ART and neurologically asymptomatic with low levels of HIV-1 RNA in plasma and elevated levels in CSF (18).

### HIV-1 genetic diversity in the CSF varies greatly across participants with NSE and can reach levels observed in the blood during chronic infection.

Single genome amplification (SGA) (36) and/or Illumina MiSeq Deep Sequencing with Primer ID (37) was used to genetically characterize HIV-1 RNA in the CSF of all 25 participants with NSE and in 4 comparator cohorts (cohorts described in Supplemental Table 2). SGA analyses yielded full-length Env glycoprotein (*env*) sequences, and MiSeq Deep Sequencing with Primer ID generated partial *env* sequences (521 bases in length) and sequences for 3 additional amplicons used to assess drug resistance (described below). We and others have previously used these approaches to assess genetic diversity in HIV-1 populations (18, 38, 39) and compartmentalization in the CSF (5, 9, 40, 41).

**Figure 1:**
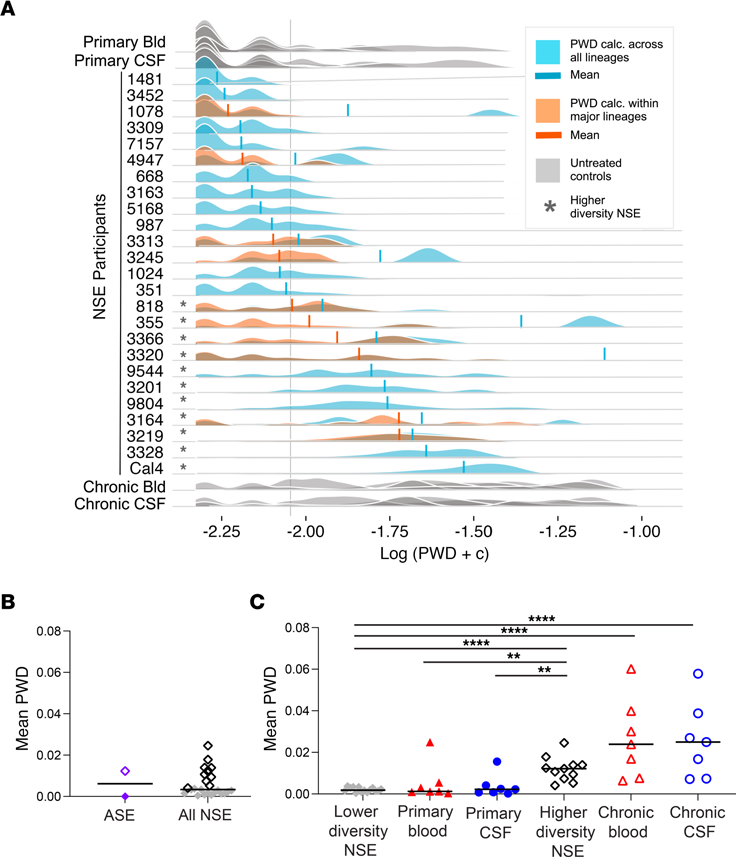
HIV-1 NSE populations can be very genetically diverse. (**A**) To facilitate plotting on a log scale, a constant (0.005) was added to all PWD values and then log transformed. PWD was calculated across all lineages in the CSF of NSE participants (blue) and untreated controls (gray). When multiple major lineages were present in a CSF NSE population, PWD was also calculated within each major lineage, thus avoiding between-lineage comparisons (orange). Means are marked with a vertical line in orange or blue. For individuals with both types of PWD calculations (shown in blue and orange), the mean value of the within-lineage comparison (orange) was used for downstream analyses. A PWD value of 0.004 roughly separated the diversity observed during primary infection from levels observed during chronic infection. The value corresponding to this cutoff is marked with a vertical gray line. Participants with mean PWD above 0.004 are marked with an asterisk. (**B**) Mean PWD was calculated for the CSF of 2 participants with ASE (PWD less than 0.004, purple closed diamond; PWD above 0.004, purple open diamond; ref. 18) and 25 participants with NSE (PWD below 0.004, gray closed diamonds; PWD above 0.004, black open diamonds). Median is shown with the horizontal bar. (**C**) Mean PWDs of lower (*n* = 14) and higher (*n* = 11) diversity NSE populations were compared with blood- (closed red triangles, *n* = 7) and CSF-derived (closed blue circles, *n* = 7) virus from untreated primary infection and blood- (open red triangles, *n* = 7) and CSF-derived (open blue circles, *n* = 7) virus from untreated chronic infection. **P < 0.001; ***P < 0.0001; ****P < 0.0001, Mann-Whitney *U* test. Median value of the mean PWDs is shown with the horizontal bar.

**Figure 2:**
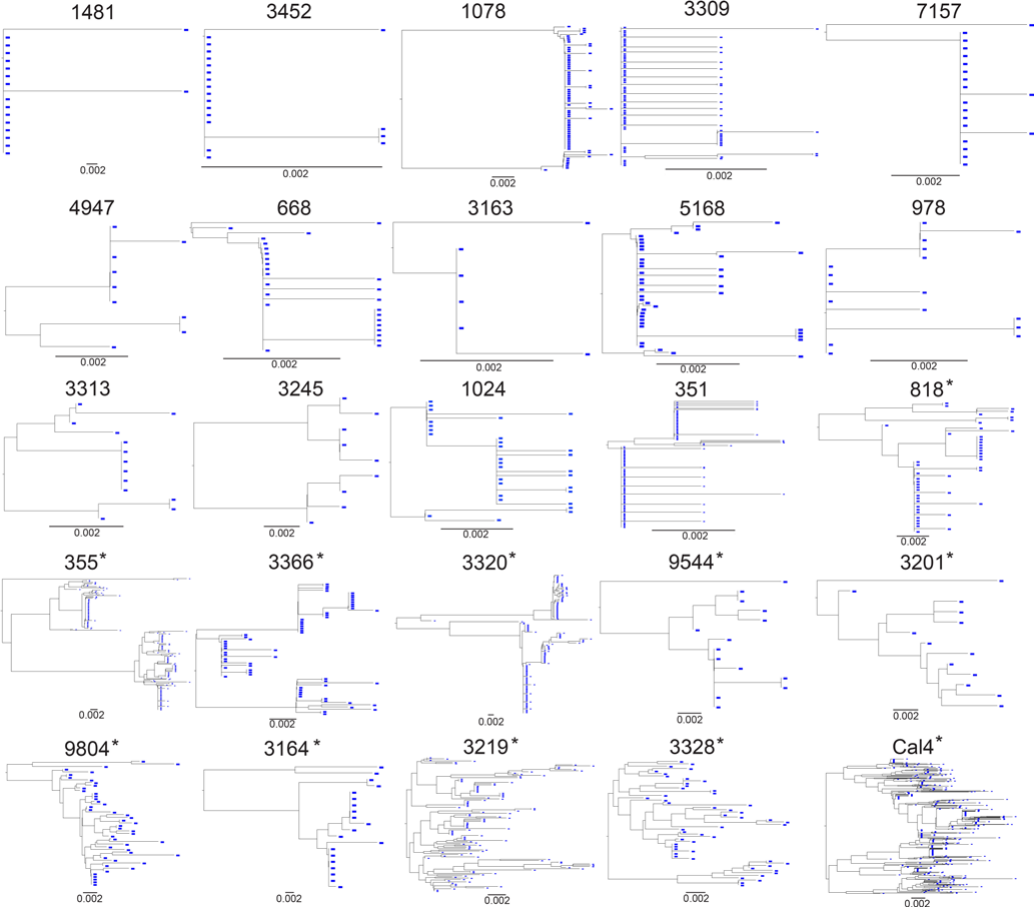
Phylogenetic trees of HIV-1 NSE populations. Genetic diversity during HIV-1 NSE was assessed using SGA and/or Illumina MiSeq Deep Sequencing with Primer ID. CSF-derived (in blue) partial *env* neighbor-joining phylogenetic trees of participants with NSE. Trees are ordered (in rows) from lowest to highest mean PWD. Asterisks designate the “higher diversity” participants with mean PWD above 0.004.

One of the primary goals of this study was to estimate the amount of diversity that accumulated in the CNS during NSE. We first calculated pairwise distance (PWD) across all lineages (Figure 1A). If NSE populations were established by multiple, genetically distinct viruses, then PWD values would represent both diversity contributed by the founders and diversity that accumulated during NSE. To minimize the contribution from founders, we identified individuals with multiple peaks, including a peak with high PWD and a phylogenetic tree with distinct major lineages (Figure 2). This is the pattern expected if an NSE population is established by multiple founder viruses. For these individuals, we recalculated PWD values within each major lineage (Figure 1A) and used the mean of those values (Figure 1A) for all subsequent analyses (when multiple major lineages were present, downstream analyses were performed using the mean of within–major lineage PWD values). While we can’t know for sure if an NSE population was founded by multiple variants, this approach reduces the impact of multiple founder viruses on PWD and generates conservative PWD estimates that better represent diversity accumulated in the CNS during NSE.

When we analyzed samples collected from untreated people (Supplemental Table 2), we observed that a mean PWD of 0.004 roughly distinguished levels of diversity observed in primary infection from those observed during chronic infection (Figure 1A). All samples collected during untreated chronic infection had a mean PWD greater than 0.004, and most samples collected during untreated primary infection (the first year after transmission) had a mean PWD below 0.004 (Figure 1A). Therefore, we used a PWD of 0.004 to separate NSE participants into lower and higher diversity groups. The lower diversity group (PWD below 0.004) had levels of diversity similar to those observed during primary untreated infection and in an individual with T cell–tropic ASE (Figure 1, B and C). In contrast, the higher diversity group (PWD above 0.004) had diversity levels similar to those observed during chronic untreated infection and in an individual with persistent, macrophage-tropic ASE (Figure 1, B and C). This somewhat arbitrary division allowed us to compare diversity during NSE to diversity observed at different stages of disease (Figure 1C) and allowed us to determine whether the inflammatory environment was related to the amount of genetic diversity in the NSE population (described below).

### HIV-1 RNA levels in the CSF are associated with more diverse NSE populations.

**Figure 3:**
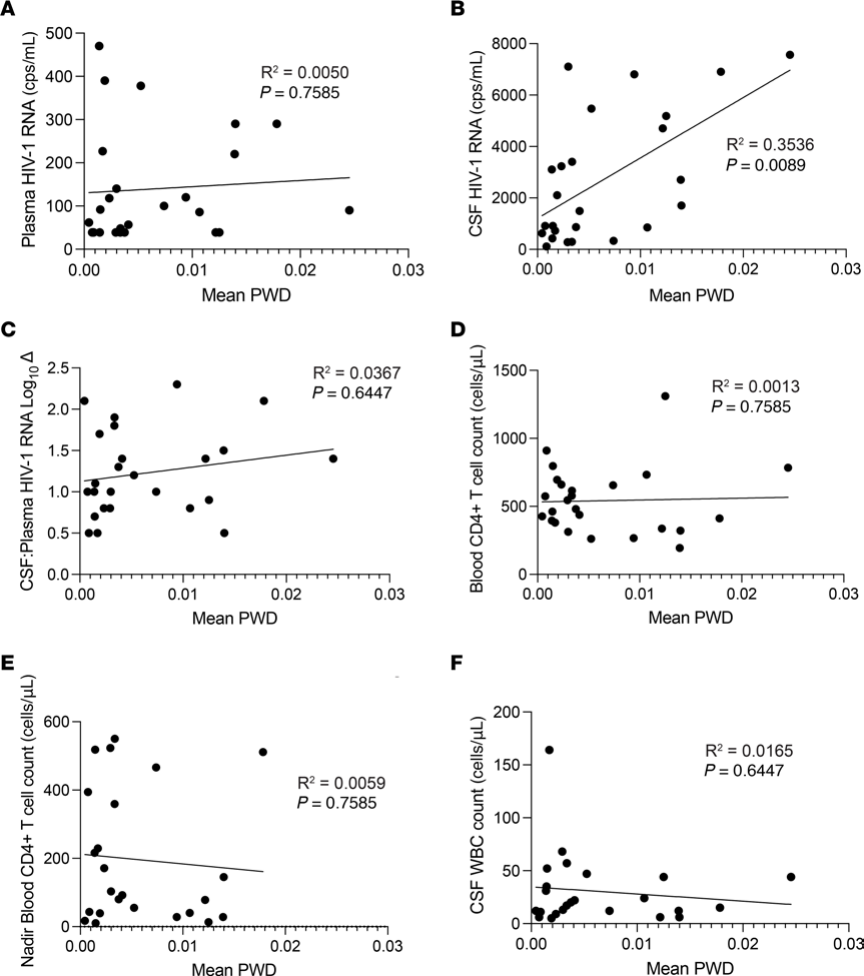
Mean PWD is correlated with CSF HIV-1 RNA. Linear regressions were performed to look for relationships between mean PWD and plasma HIV-1 RNA (**A**), CSF HIV-1 RNA (**B**), CSF: plasma HIV-1 RNA log_10_ Δ (**C**), blood CD4^+^ T cell count (**D**), nadir blood CD4^+^ T cell count (**E**), and CSF WBC count (**F**). The relationship between mean PWD and CSF HIV-1 RNA (**B**, *R^2^* = 0.3536, *P* = 0.0089) was the only statistically significant comparison. All *P* values were adjusted for multiple comparisons.

PWD levels were not significantly associated with plasma HIV-1 RNA, CSF:blood HIV-1 RNA log_10_ Δ, CSF WBC count, or blood CD4^+^ T cell count at the time of escape, but were associated with higher HIV-1 RNA levels in the CSF (Figure 3). Similarly, when NSE participants were grouped by diversity, the median of the mean PWD estimates in the higher diversity group was significantly greater than that of the lower diversity group (Supplemental Figure 1; Mann-Whitney *U* test, *P* < 0.0001).

A subset of participants (23 of 25, Supplemental Table 1) in the NSE cohort were tested for CNS coinfections as part of clinical care and/or a research study (42). Analysis of CSF samples revealed that 9 of 23 people were positive for 1 or more agents, with 8 having detectable EBV in the CSF. Since EBV was the primary coinfecting agent identified in this cohort, we determined whether EBV was related to HIV-1 levels and/or inflammation in the CNS during NSE. We divided the 18 people tested for EBV into those who were positive (*n* = 8) and those negative (*n* = 11) for EBV. EBV coinfection was not significantly associated with HIV-1 RNA in the plasma or CSF, CSF:blood HIV-1 RNA log_10_ Δ, mean PWD, blood CD4^+^ T cell count at the time of escape, nadir CD4^+^ T cell count, and/or CSF WBC count (Supplemental Figure 2).

### Virus in the CSF during NSE is typically resistant to some, but not all, drugs in the ART regimen.

In this study, drug resistance was assessed based on partial integrase, protease, and reverse-transcriptase sequences generated by Illumina MiSeq Deep Sequencing with Primer ID (37). A total of 91% (21/23) of participants analyzed were at least partially resistant to their current ART regimen, but only 15% (4/23) had a resistance mutation to all drugs in their regimen (Table 2). Further, of the 5 participants in this cohort with longitudinal sampling (Table 3), 1 participant (no. 351) was observed to lose and acquire drug-resistance mutations during the study. The complex changes in drug resistance in this participant are consistent with viral replication in the CNS in the presence of low drug levels and a period of treatment interruption (See Table 3).

### NSE typically resolves if ART is changed to a regimen to which the CSF viral population is sensitive (i.e., ART optimization).

ART optimization was typically associated with reductions in HIV-1 RNA in the CSF and improved neurologic symptoms (Supplemental Table 3). In contrast, when ART was not optimized, drug-resistant NSE virus persisted in the CNS (Table 3 and Supplemental Figure 3). Given that ART stops viral replication, but not viral production, these results suggest that during NSE, HIV-1 RNA in the CSF is being produced by replication that contributes to neurologic symptoms. One limitation of this interpretation is that not all follow-up visits took place soon after ART optimization.

### NSE virus is adapted to replication in CD4^+^ T cells.

**Figure 4:**
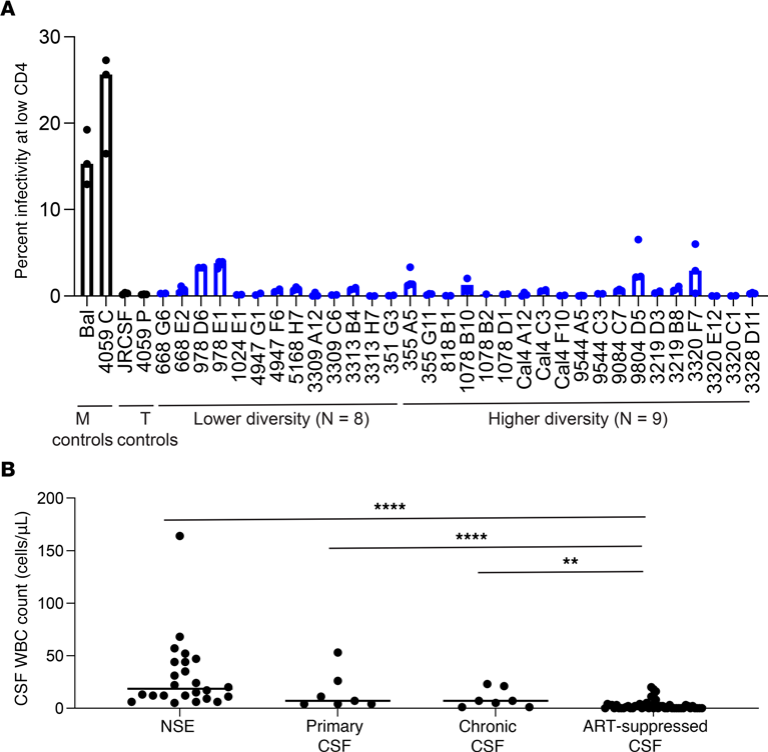
NSE virus is adapted to replicating in CD4^+^ T cells. (**A**) Full-length CSF-derived HIV *envs* generated by SGA were cloned and used to produce pseudoviruses that were then tested in our Affinofile assay for their ability to facilitate entry into cells with low levels of CD4. None of the NSE *envs* tested were able to efficiently enter cells with low levels of CD4, but rather required high surface density of CD4 for efficient entry, indicating that the virus was adapted to replicating in T cells. Median relative infectivity values for 2 to 4 biological replicates are plotted with bars, and each individual point is shown with a circle. Representative CSF *envs* from both the lower diversity group (*n* = 8) and the higher diversity group (*n* = 9) were tested (blue bars and circles) as well as macrophage-tropic and T cell–tropic controls (black bars and circles). (**B**) CSF WBC counts in the NSE cohort and each comparator cohort. Mann-Whitney *U* tests were performed and found the ART-suppressed cohort (*n* = 49) had significantly lower CSF WBC counts than participants with NSE (*n* = 25), untreated primary infection (*n* = 7), and untreated chronic infection (*n* = 7): ****P = 0.0002; ****P = 0.0002; **P = 0.0044, respectively. All *P* values were corrected for multiple comparisons. Median values are shown with horizontal bars.

HIV-1 replication in the CNS can take place in resident or trafficking CD4^+^ T cells (4, 9, 18, 43) or in resident CD4^+^ macrophage or microglia (4, 18). Using Affinofile cells (44) and our established protocol (45), we assessed the ability of NSE virus to enter cells with low levels of CD4 on the surface. We tested 32 patient-derived *envs* from 17 NSE participants with different levels of diversity in the NSE population and all were found to require a high density of CD4 for entry (Figure 4A), similar to the levels on CD4^+^ T cells (46), and were unable to efficiently infect cells expressing low CD4 levels like those found on macrophage/microglia. Thus, we conclude that the virus detected in the CSF during NSE is adapted to replication in CD4^+^ T cells. Further, the frequency of WBCs in the CSF was elevated during NSE relative to levels found in people on suppressive ART (Mann-Whitney *U* test, *P* < 0.001; Figure 4B).

### CSF biomarkers of inflammation are elevated in NSE.

**Figure 5:**
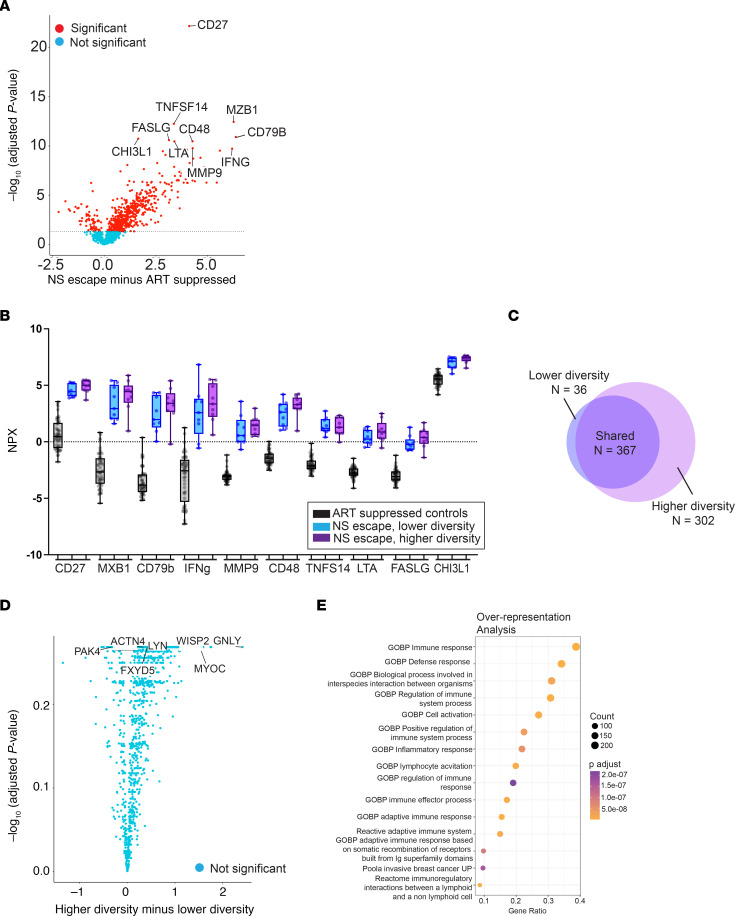
Inflammatory biomarkers in the CSF are elevated in participants with NSE compared with ART-suppressed controls and trend higher in NSE participants with high-diversity viral populations compared with NSE participants with low-diversity viral populations. We measured 1,472 protein biomarkers (Olink platform) in CSF of 18 NSE participants and 49 ART-suppressed controls. (**A**) Volcano plot comparing CSF biomarker levels during NSE to levels in suppressed controls. Levels of 678 biomarkers were significantly different after correction for multiple comparisons (red circles, most higher in NSE). The 10 biomarkers with the lowest adjusted *P* values are labeled. (**B**) Distributions (quartiles) of these 10 biomarkers are shown. (**C**) Venn diagram of the 705 CSF biomarkers that are significantly different between NSE participants and suppressed controls. (**D**) Volcano plot comparing CSF biomarkers in participants with lower versus higher diversity NSE populations. No markers were significantly different, but there was a trend for the CSF biomarkers to be higher in participants with higher diversity NSE (majority of circles to the right of 0 on the *x* axis). (**E**) Overrepresentation analysis of gene ontogeny biological processes. The 14 most overrepresented pathways in the CSF of NSE participants compared with ART-suppressed controls are shown. The color of each circle indicates the adjusted *P* value, and the size of the circle represents count. We found that 40% of the genes that were overrepresented in the CSF of NSE participants compared with ART-suppressed controls were involved in the immune response (top row).

We used the Olink Explore 1536 platform to measure a large panel (*n* = 1,472) of proteins in CSF. We compared proteins in the CSF of a subset (*n* = 18/25) of NSE participants to those of ART-suppressed participants with undetectable HIV-1 RNA in the CSF and plasma while on suppressive therapy (*n* = 49, Supplemental Table 2). This analysis revealed that 48% (705/1,472) of all biomarkers analyzed were higher in the CSF during NSE compared with levels in the CSF during controlled infection (*t* test with FDR correction; Figure 5A).

### The higher genetic diversity in the NSE population was associated with greater inflammation.

Next, we focused on the 10 biomarkers that differed most significantly between the ART-suppressed participants and NSE participants (i.e., the 10 lowest *P* values) and explored whether the lower and higher diversity groups differed for those markers (Figure 5B; diversity groups defined in Figure 1A). We found that biomarker levels were significantly elevated in both the lower and higher diversity groups compared with the ART-suppressed group and biomarker levels in the higher diversity group tended to be greater than those in the lower diversity group, though this trend was not statistically significant (Supplemental Table 4).

We then performed a comprehensive analysis of the full biomarker panel. A total of 705 CSF biomarkers were significantly different in the NSE participants compared with ART-suppressed controls (Figure 5C). The lower diversity group had 403 biomarkers that differed from the ART-suppressed controls, and 91% (367/403) of these differences were shared with the higher diversity group. In contrast, the higher diversity group differed from ART-suppressed controls in 669 markers and only 55% of these differences were shared with the lower diversity group (367/669; Figure 5C). To examine the directionality of these differences, we performed a differential expression analysis comparing the 2 NSE groups and found that CSF biomarker levels tended to be elevated in the higher diversity group relative to the lower diversity group (Figure 5D). While differences in individual proteins did not reach statistical significance, overall, a greater number of proteins were elevated in the higher diversity group relative to the lower diversity group (Fisher’s exact test, *P* < 0.0001). To determine the gene pathways in which the upregulated proteins were involved, we performed an overrepresentation analysis (R package OlinkAnalyze). This analysis revealed that 40% of the biomarker genes overrepresented in the CSF of NSE participants are involved in immune responses (Figure 5E). These results indicate that the immune environment is dysregulated during NSE, but the number of biomarkers involved and the magnitude of dysregulation are greater in the higher diversity group than in the lower diversity group. This suggests that NSE may be a progressive state and that higher diversity NSE is the more advanced form.

### HIV-1 populations in the plasma during NSE are genetically similar to those in the CSF.

A subset of NSE participants (*n* = 9) had a plasma HIV-1 RNA below 500 copies/ml, but high enough to make sequencing possible. We sequenced virus populations in blood and CSF of these individuals. The presence of genetically distinct lineages in the CSF and blood (i.e., compartmentalization) would indicate that virus populations were replicating independently in the 2 compartments. In all 9 participants, virus populations in the blood plasma and CSF were equilibrated at the time of NSE (representative trees in Supplemental Figure 4), indicating that independent replication was not taking place in both compartments. Additionally, drug-resistance profiles measured by Illumina MiSeq with Primer ID of virus in the blood plasma matched the drug-resistance profiles in virus in the CSF at the time of escape.

## Discussion

We sought to understand the source of HIV-1 RNA in the CSF of 25 PLWH and experiencing NSE. These participants had elevated HIV-1 RNA in the CSF and severe neurologic symptoms. There are 2 likely mechanisms by which HIV-1 RNA could accumulate in the CSF during otherwise suppressive ART. First, infected cells in the CNS could express virus that does not replicate due to ART (i.e., virus expression/production without replication). Alternatively, drug-resistant virus could replicate in the CNS. Here, we explored which of these mechanisms (virus expression/production or replication) generates HIV-1 RNA in the CSF during NSE. While NSE has previously been studied (see refs. 12, 13, 47–51), we believe this is the first detailed genetic and phenotypic analysis of NSE virus and its association with ongoing CNS pathogenesis during ART.

**Figure 6:**
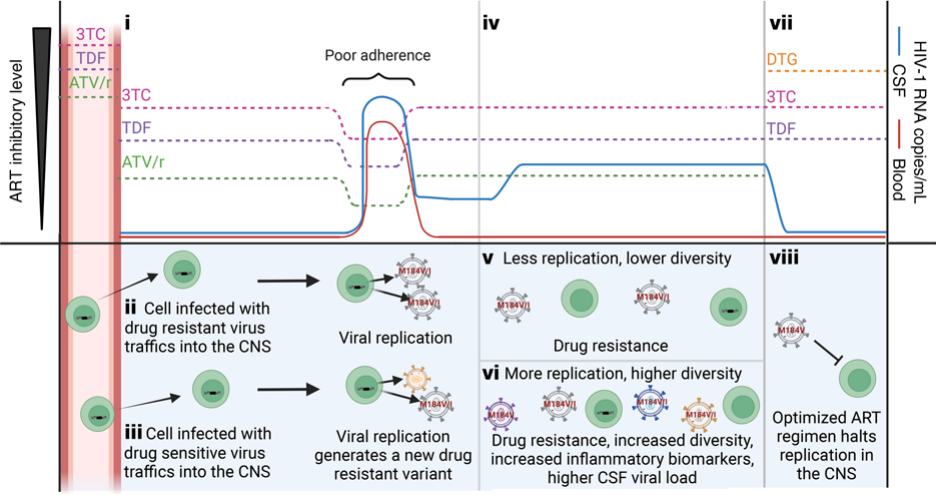
Model of the emergence of NSE during treatment with an ART regimen representative of those used in this cohort. (i) Most drugs are at lower concentrations in the CSF (86). Despite the overall low levels of protein in the CSF, most ATV in the CSF is protein bound (54), thus reducing the amount of available ATV (54) to noninhibitory levels (86). In contrast, TDF and 3TC are primarily unbound in the CSF. In this drug regimen (TDF/3TC/ATV/r, the most common regimen in our cohort), 3TC is likely the only drug at inhibitory levels in CSF. During a period of poor adherence, the concentration of drug falls and the CSF HIV-1 RNA rises. Upon returning to adherence, drug levels rise and HIV-1 RNA decreases. Return to adherence will select for the M184V mutation (conveying resistance to 3TC). (ii) Resistant virus can reach the CNS as a migrating CD4^+^ T cell carrying a provirus with the M184V mutation or (iii) evolve in the CNS during replication when all 3 drugs are at noninhibitory levels. (iv) HIV-1 RNA in the CSF remains detectable and may increase as the drug-resistant virus replicates. (v and vi) Replication increases genetic diversity, CSF HIV-1 RNA, and inflammatory biomarkers. Both v and vi are associated with the spread of drug resistant virus and an elevation in CSF WBC. (vii) Optimization of ART to a regimen with good CNS penetration and to which the NSE virus is sensitive will (viii) stop replication and improve symptoms. Created with BioRender.

Genetic analyses in this study yielded 4 observations indicating that virus found in the CSF during NSE is produced by HIV-1 replication. Below we discuss these observations and present a model of how NSE likely emerged in participants on the most common ART regimen in this cohort (lamivudine [3TC]/tenofovir disoproxil fumarate [TDF]/ATV/ ritonavir [/r]) (Figure 6).

The first observation indicating that NSE is produced by viral replication is that virus in the CSF during NSE is typically resistant to some, but not all, drugs in the ART regimen. This has also been observed in previous studies (12, 13, 49, 50–53). Importantly, we observed that virus in the CSF during NSE is always resistant to drugs found at reasonably high concentrations in the CNS (e.g., nucleoside reverse transcriptase inhibitors [NRTIs]), but is often sensitive to drugs found at lower concentrations in the CNS (e.g., the PI ATV). This is consistent with NSE being produced by virus replication and adaptation to the unique drug environment of the CNS.

Based on the results of this study, we have developed a model of how drug-resistant virus likely emerged in participants on the most common ART regimen in this NSE cohort (3TC/TDF/ATV/r) (Figure 6). This regimen includes 2 drugs (TDF/ATV) that may be found at suboptimal levels in the CSF (54, 55) and a third drug (3TC) that is typically above the WT IC_50_ (56) (Figure 6, i). The low CSF concentration of ATV has been extensively studied (see refs. 53, 54) and was previously associated with CSF escape (50, 57). In our NSE cohort, all individuals on this regimen (3TC/TDF/ATV/r) had the same mutation (M184V/I), which provided resistance (58, 59) to the drug in the regimen (3TC) most likely to be at inhibitory levels in the CSF. Drug-resistant virus could have trafficked into the CNS (Figure 6, ii), or if adherence was poor, M184V/I could have evolved while HIV-1 was replicating in the CNS (Figure 6, iii). Once ART adherence was reestablished and 3TC returned to inhibitory levels, the drug-sensitive variants would have been unable to replicate (Figure 6, iv). Regardless of its origin, the M184V/I mutation could have facilitated replication and spread in the CNS if 3TC were the only drug at high levels. Interestingly, this resistance mutation increases susceptibility to TDF (60, 61). This provides additional evidence that M184V/I mutants were selected in environments where TDF levels were low due to either poor penetrance or poor adherence.

The second observation indicating that the NSE virus is produced by replication comes from changes in drug-resistance profiles that we observed in a participant with NSE. Most drug-resistance mutations reduce fitness in the absence of the drug (reviewed in ref. 62), making it unlikely that they spread due to chance (i.e., genetic drift), but more likely that they spread due to natural selection during replication in the presence of drug. Longitudinal analyses performed on a subset of participants in this cohort identified 1 participant (no. 351) whose drug-resistance profile changed during NSE. The complex pattern of loss and acquisition of drug-resistance mutations in the CSF of this participant is consistent with viral replication in the CNS in the presence of low drug levels and a possible period of treatment interruption.

The third observation indicating that NSE virus is produced by replication is the fact that some people with NSE had very diverse HIV-1 populations in their CSF. The amount of genetic diversity in an NSE population may be higher if the population was founded by multiple variants (e.g., multiple, genetically distinct, drug-resistant viruses) and/or the population replicated and evolved during NSE. To assess the amount of diversity generated by replication and evolution during NSE and minimize the contribution that multiple founders made to diversity, we calculated genetic diversity within (not between) major lineages. A limitation of this approach is that an NSE population could be founded by multiple variants, but over time, recombination and evolution could erase evidence of those distinct founders. While this situation would cause us to overestimate the amount of diversity generated by replication during NSE, this issue would only arise when there is extensive virus replication during NSE.

After minimizing the impact of founders on estimates of viral diversity in NSE populations, we observed that some NSE populations had very high levels of diversity like those observed during untreated chronic infection. This pattern suggests that for some people with NSE, diversity likely accumulated over many rounds of replication in the CNS (compare Figure 6, v and vi). The only way that very high levels of genetic diversity could be observed without virus replication is if a large number of CNS cells infected with genetically distinct, drug-resistant proviruses were expressing those proviruses at the same time — an unlikely event.

The fourth observation indicating that NSE virus is produced by replication is the observation that optimization of ART (Figure 6, vii) was associated with suppression of HIV-1 RNA in the CSF (Figure 6, viii). After initiating optimized regimens to which NSE virus was sensitive, 93% (13/14) of participants in this cohort saw a reduction in CSF HIV-1 RNA (Supplemental Table 3) and all participants had improvement in neurologic symptoms/complaints. Similarly, other studies have also found that NSE resolves after treatment optimization (49, 63). In the absence of replication, ART optimization would have no effect on HIV-1 RNA levels in the CSF. Consistent with this, previous studies have observed that NSE can remerge when adherence is poor or ART is modified to a regimen to which the NSE virus is resistant and/or a regimen that does not efficiently penetrate the CNS (49, 64). Together, the 4 genetic patterns highlighted in the current study indicate that NSE is produced by viral replication in the CNS during ART.

It is notable that replication of drug-resistant NSE virus in this study produced CSF HIV-1 RNA levels that were lower than those typically observed in the CSF during untreated infection. There are a number of mechanisms that may limit viral replication in the CNS during ART. First, drug resistance typically reduces replicative fitness (reviewed in ref. 62). For example, in the presence of drug, fitness of HIV-1 with the M184V/I mutation is approximately 10% that of WT virus in the absence of drug (65). In addition, while some drugs may not reach completely inhibitory levels in the CSF, they may reach levels that slow virus replication. Finally, prior to ART initiation, CSF virus is usually closely related to virus in the blood, suggesting that virus in the CSF is produced by actively infected cells frequently trafficking into the CNS and releasing large amounts of virus (see ref. 66). However, during ART, most CD4^+^ T cells in the periphery are either infected with defective virus (67) or are latently infected (67) and unlikely to release large amounts of virus into the CSF if they traffic to that compartment. Together, these factors may slow viral replication during NSE, reduce virus production by trafficking cells, and explain why CSF HIV-1 RNA levels during NSE are typically lower than those observed during untreated infection.

There is now strong evidence that NSE is produced by replication in CD4^+^ T cells in the CNS during ART. The CNS is an immune-privileged site where total CSF WBCs, and thus CD4^+^ T cells, are typically low in PLWH and people on suppressive ART, but are greatly elevated during NSE (68). We observed that all viruses isolated from the CSF during NSE were well adapted to infecting CD4^+^ T cells, but not macrophages (macrophage/microglia are the other target cell for HIV-1 in the CNS). This indicates that these HIV-1 variants had been replicating in CD4^+^ T cells during NSE. This is consistent with a recent study that used immunoprecipitation to pull down virus in the CSF during NSE and found that CD26, a marker typically observed on CD4^+^ T cells but not macrophages, was incorporated into a high percentage of virions, indicating virus in the CSF during NSE had budded from CD4^+^ T cells (69).

Our results suggest that NSE is a progressive state in which diversity and inflammation increase as HIV-1 replicates in the CNS. This is supported by our observation that people with more diverse NSE populations had a larger number of elevated biomarkers and higher CSF HIV-1 RNA levels than people with lower diversity NSE populations. It is worth noting that, while replication is likely the force driving NSE, inflammation may also affect symptoms. This is consistent with a study showing that HIV-1 RNA levels in the CSF declined when a person on ART with progressive multifocal leukoencephalopathy immune reconstitution inflammatory syndrome (PML-IRIS) and drug-resistant HIV-1 in the CNS was treated with corticosteroids (70). Thus, we cannot rule out the possibility that an inflammatory environment in the CNS increases HIV-1 diversity during NSE by either bringing in HIV-infected cells from the periphery or enhancing virus replication in the CNS. However, our observation that in this cohort, ART optimization is associated with both suppression of virus in the CSF and improved symptoms provides strong evidence that replication is driving pathogenesis in people experiencing NSE.

We also explored whether a CNS coinfection could have affected HIV-1 replication in our NSE cohort. EBV was the only CNS coinfection identified in a substantial proportion of participants, with 35% having detectable EBV DNA in CSF. Detection of EBV DNA in CSF has previously been associated with higher CSF WBC counts (71), CSF escape in people on ART (72), and higher HIV-1 RNA in the CSF during untreated infection (72). We did not identify any differences in CSF WBCs and/or HIV-1 RNA in CSF between people with and without EBV DNA in CSF. Similarly, detection of EBV DNA in CSF was not associated with differences in HIV-1 genetic diversity in the CSF during NSE. Together, these observations suggest that the presence of EBV DNA in the CSF did not substantially impact viral replication during NSE.

Our findings suggest that NSE is very different from other types of persistent viremia observed in people on ART, including ASE. Both NSE and ASE are defined by elevated HIV-1 RNA in the CSF of an otherwise well-suppressed person, but only NSE is associated with overt neurologic symptoms. Since there are no obvious symptoms associated with ASE, it is primarily identified in the context of research studies (not patient care), where it has been observed in 5%–15% of people on ART (34, 35, 73–76), making ASE much more common than NSE. Longitudinal analyses suggest that, like “blips” in the plasma during ART, ASE is typically transient (34). ASE is also presumably benign based on observed low levels of CSF WBCs (77, 78), CSF inflammatory biomarkers, and CSF neurofilament light chain (NFL) (a marker of neuronal damage) in people with ASE (34, 77, 78). In contrast, NSE often persists until ART is optimized (this study and refs. 13, 68) and is associated with very high CSF WBC counts (this study and refs. 12, 13, 68, 77, 79). NSE is also associated with extremely high levels of inflammatory CSF biomarkers and CSF NFL that are similar to levels observed in untreated people with HAD (77).

Differences in the pathogenesis of ASE and NSE are likely because the 2 types of CSF escape are generated by different mechanisms. In a previous study of 2 people with ASE (18), we found that ASE can be produced by either replication in macrophage or virus expression from CD4^+^ T cells in the absence of replication (18). It is currently unknown how often ASE is generated by each mechanism. Given that most CD4^+^ T cells expressing HIV-1 RNA have a short half-life (see ref. 80), expression from short-lived CD4^+^ T cells would explain why ASE is transient, but this needs further study. In contrast, here we show that NSE is produced by replication of HIV-1 in CD4^+^ T cells in the CNS.

The fact that NSE is produced by replication in CD4^+^ T cells also sets it apart from nonsuppressible viremia (NSV) observed in the plasma during ART. Studies of NSV indicate that it is typically produced by expression of defective proviruses in CD4^+^ T cells (28, 81). Interestingly, NSV and ASE are not generated by replication in CD4^+^ T cells during ART and have no overt pathogenic effects. This raises the possibility that sustained viral replication in CD4^+^ T cells during NSE generates pathogenic effects that are far more severe than those generated by proviral expression (source of NSV and T cell–tropic ASE) and/or low-level replication in macrophages (the source of macrophage-tropic ASE).

### Summary.

Neurologic symptoms are some of the most common comorbidities in PLWH on ART. A minority of people experiencing such symptoms have NSE (see ref. 63). While rare, NSE continues to be a problem for PLWH, particularly in resource-limited settings where access to a variety of ART regimens is limited and clinical follow-up is infrequent. Our results suggest a model (Figure 6) in which HIV-1 replication in CD4^+^ T cells in the CNS during ART contributes to inflammation and neurologic symptoms during NSE. The ability of HIV-1 replication in CD4^+^ T cells to drive neuropathogenesis is also supported by studies observing evidence of sustained HIV-1 replication in CD4^+^ T cells in the CNS of some untreated individuals with HAD (4, 40). This represents a mechanism generating HIV-1 RNA during ART that is distinct from mechanisms generating ASE in CSF and NSV in plasma. While optimization of ART can stop viral replication during NSE, a pool of latently infected CD4^+^ T cells may persist in the CNS and reemerge if adherence is poor and/or the drug regimen is altered (49). This contributes to the growing body of evidence that CD4^+^ T cells in the CNS are major targets of HIV-1 replication and that replication in these cells before or during ART generates CNS inflammation and neurologic symptoms.

## Methods

### Sex as a biological variable.

Of the 25 participants enrolled in this study, 10 were assigned as female at birth. Data analyses were performed to determine whether study results were related to sex.

### Study design.

For the main NSE cohort, archived CSF and blood plasma were identified from patients who were HIV^+^, on ART, presenting with overt neurological symptoms and signs, and experiencing discordant elevations in CSF HIV-1 RNA. Samples were collected between 2000 and 2019 in 4 countries: Pune, India (*n* = 12); San Francisco, California, USA (*n* = 7); Milan, Italy (*n* = 3); Torino, Italy (*n* = 1); and Gothenburg, Sweden (*n* = 2). For the chronically infected cohort, 7 untreated, chronically infected PLWH were used as comparators for sequence diversity and mean PWD. For the primary HIV infection cohort, 7 untreated PLWH sampled during primary HIV infection (within the first 12 months of infection) were used as comparators for sequence diversity and mean PWD. For the Olink control cohort, 49 ART-suppressed PLWH participants were used as controls for the Olink biomarker analyses.

### Sample collection and HIV-1 RNA analyses.

Whole blood was collected in EDTA, and the plasma was separated from whole blood pellet by centrifugation at 800*g* and 4°C and then stored in aliquots at –80°C. CSF was collected, centrifuged to pellet cells at 800*g* and 4°C, and the supernatant was stored in aliquots at –80°C. HIV-1 RNA levels were measured in blood plasma and CSF using the Abbott RealTime HIV-1 RNA Assay (lower limit of quantification = 40 copies/ml) or COBAS Ampliprep/COBAS TaqMan, version 2.0 (lower limit of quantification = 20 copies/ml).

### SGA.

SGA was performed as previously described (36) (primer sequences are shown in Supplemental Table 5). Briefly, viral RNA was extracted from blood plasma and CSF, and cDNA was generated using an Oligo dT_20_ primer (Invitrogen). The cDNA was diluted to end-point to minimize the possibility of recombination, and semi-nested PCR was performed. Full-length *envs* were bidirectionally sequenced using Sanger sequencing or PacBio SMRT sequencing with barcodes. Envs were then aligned using multiple sequence comparison by log-expectation (MUSCLE) (82). Neighbor-joining phylogenetic trees were generated with MUSCLE and visualized using FigTree, version 1.4.4, software (http://tree.bio.ed.ac.uk/software/figtree/).

### Cloning and generation of pseudovirus.

SGA-generated *envs* to be cloned were selected based on each phylogenetic tree structure. Each full-length *env* chosen was cloned into a pcDNA3.1D/V5-His-TOPO expression vector (Invitrogen) using the pcDNA3.1 TOPO Expression Kit (Invitrogen). Env-pseudotyped luciferase reporter viruses were generated as previously described (41). Briefly, 293T cells were cotransfected with an *env* expression vector and the pNL4-3.LucR-E-HIV-1 backbone (NIH Reagent Program) using the Fugene HD transfection reagent protocol (Promega).

### Affinofile entry assay.

We used our established protocol (46) to assess the ability of these reporter viruses to enter cells expressing a low density of CD4 (a marker for macrophage tropism). Briefly, pseudoviruses were titered on CD4^hi^CCR5^hi^ cells to ensure the infection assay was performed within the linear range. Affinofile cells (44) were then induced to CD4^hi^CCR5^hi^ and CD4^lo^CCR5^hi^ and cells under both conditions were infected with 800,000 RLUs of pseudovirus to determine the ability of cloned *envs* to facilitate entry into cells with a low density of CD4.

### Deep sequencing.

Illumina MiSeq 300 base paired-end multiplex library preparation was done using our primer ID method, which avoids resampling and allows for the correction of errors introduced during PCR and sequencing (37). Briefly, viral RNA was extracted from blood plasma and CSF, and cDNA was generated using a pool of 4 primers to generate amplicons for the V1–V3 region of *env* and partial IN, RT, and PR regions of pol (Supplemental Table 6). Each cDNA primer included a random 11 base tag as the primer ID. cDNAs were amplified and sequenced using the Illuminia MiSeq library prep and sequencing platform (primer sequences are shown in Supplemental Table 5). A template consensus sequence (TCS) was generated for each primer ID, and TCSs were aligned using MUSCLE (82). Neighbor-joining phylogenetic trees were then generated with MUSCLE and visualized using FigTree, version 1.4.4, software. Full-length *env* sequences generated by SGA were incorporated into Illumina MiSeq V1V3 deep-sequencing trees by first trimming full-length envs to the 521 bp MiSeq amplicon, then aligning the trimmed sequences with the MiSeq sequences (MUSCLE) and generating neighbor-joining phylogenetic trees.

### Olink explore methods.

CSF protein biomarker measurements included in this analysis were part of a larger study measuring protein concentrations across 307 CSF specimens from a broad spectrum of HIV-1 infection along with HIV-uninfected controls. For this analysis, samples from 4 clinical sites (Gothenburg, San Francisco, Pune, and Milan) were used. These were maintained at –70°C, aggregated in San Francisco, and shipped together to Olink Laboratory (Watertown, Massachusetts, USA) in previously stored, frozen aliquoted tubes and analyzed in November 2020. Samples were transferred using local Olink procedures, with each sample Explore 1536 battery deployed at that time consisting of 4 plates: inflammation, cardiometabolic, oncology, and neurology plates. The OLINK immunoassays are based on proximity extension assay (PEA) technology (83), which uses a pair of oligonucleotide-labeled antibodies to bind to their respective target proteins. When the 2 antibodies are in close proximity, a new PCR target sequence is formed, which is then detected and quantified by real-time PCR. The concentration ranges for measurement of each protein are reported in NPX log_2_ units, which provide relative concentrations that are used for within-assay comparison of sample concentrations (https://olink.com/).

### Statistics.

PWDs were calculated using MEGAX software, version 10.1.8 (84, 85). For participants with bimodal PWD distributions, mean PWD was calculated by taking the mean PWD of each major lineage and then calculating the mean of those values. For participants without a bimodal PWD distribution, the overall mean was used. *t* Tests with FDR corrections for the differential expression analyses of biomarkers were done using R statistical software, version 4.2.1, and all other Mann-Whitney rank-sum tests were done using GraphPad Prism, version 9.1.2, software. Overrepresentation analyses for biomarker data were done with the R package OlinkAnalyze using R statistical software, version 4.2.1. Adjusted *P* values of less than 0.05 were considered significant.

### Study approval.

This study was approved by the IRBs at the University of North Carolina at Chapel Hill (IRB protocol 15-2082), the University of California at San Francisco (IRB protocols 10-0727 and 10-01171), the University of Gothenburg (IRB protocol O588-01), the University of Torino (IRB protocol 198/2105), the San Raffaele Scientific Institute (IRB protocol CE 235/2015), and the Poona Hospital and Research Center. All specimens were collected in the context of research protocols approved by local IRBs. All participants were adults (≥18 years of age), and written, informed consent was obtained.

### Data availability.

The sequences of the full-length *env* amplicons are available in GenBank (OR736341–OR736607). The deep sequencing data are available in the Sequence Read Archive (BioProject ID PRJNA1065861). Illumina MiSeq data were analyzed using TCS pipeline, version 2.5.1 (https://www.primer-id.org/?from_old). Olink raw data and analysis scripts are available here: https://github.com/ViralSeq/NS-escape-Olink/tree/3a75d5fe33d10d24160ef21ee5dafcfb8b421682; commit ID 3a75d5f. Values for all data points in graphs are reported in the Supporting Data Values file.

## Author contributions

SBJ, RWP, MG, PC, and SS conceived and designed the study. AD, PC, MT, AC, MG, RV, and RWP provided clinical samples. LPK conducted the experiments. LPK, SZ, SS, MG, RWP, and SBJ analyzed the data. LPK and SBJ wrote the manuscript with input from AD, PC, MT, AC, SZ, SS, MG, and RWP.

## Supplementary Material

Supplemental data

Supporting data values

## Figures and Tables

**Table 1:**
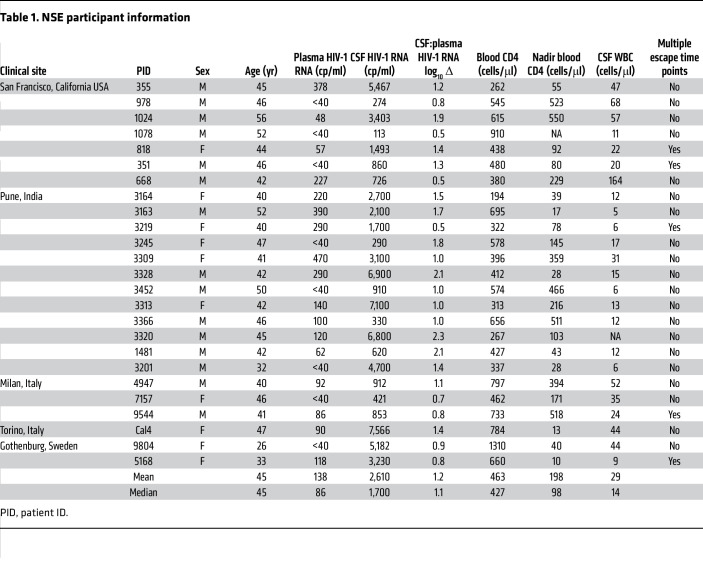
NSE participant information

**Table 2:**
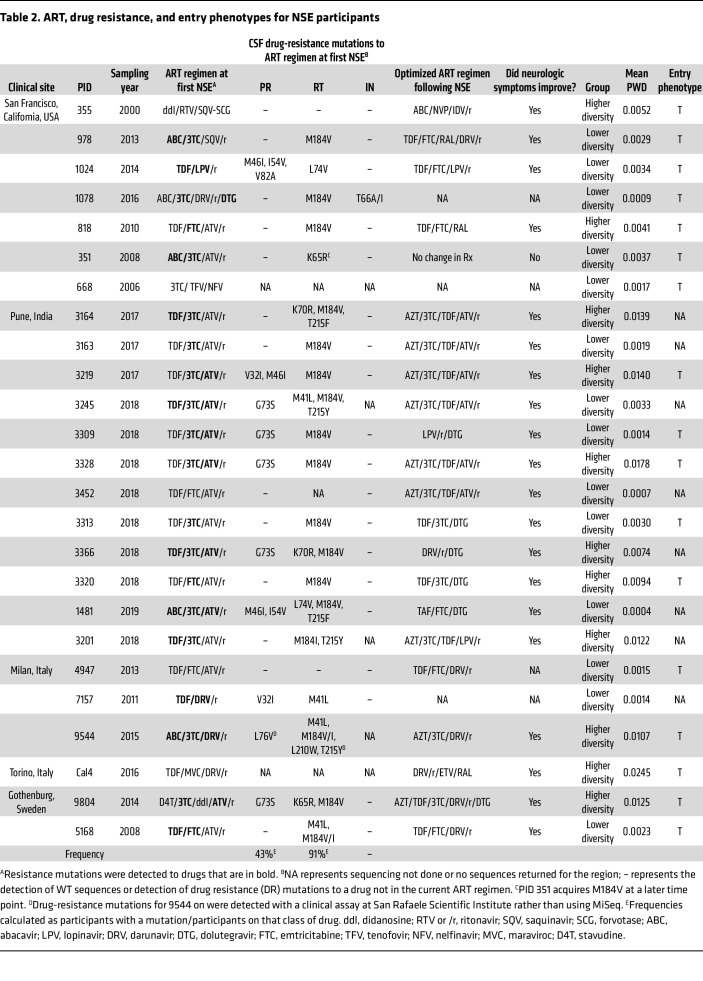
ART, drug resistance, and entry phenotypes for NSE participants

**Table 3:**
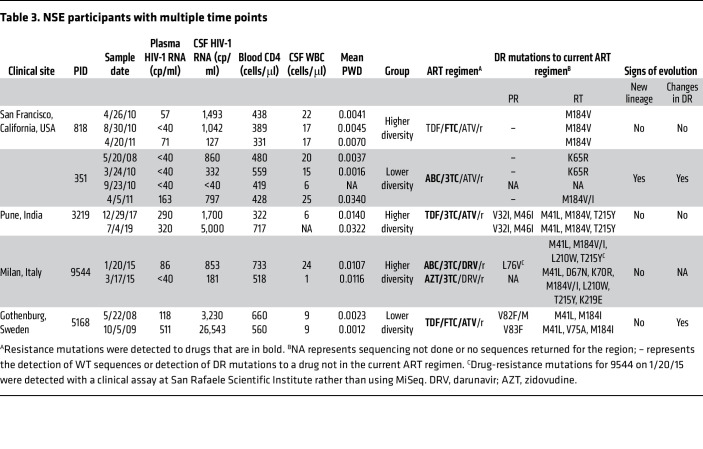
NSE participants with multiple time points

## Version 1 10/01/2024

Electronic publication
