# Supplementary material for: Neurosymptomatic HIV-1 CSF escape is associated with replication in CNS T cells and inflammation
Source: J Clin Invest. 2024 Oct 1;134(19):e176358. doi: 10.1172/JCI176358 (PMC11444166; doi:10.1172/JCI176358)
Supplement: Supplemental data [file jci-134-176358-s094.pdf]

Supplemental Table 1. Clinical symptoms and coinfections

Clinical site	PID	Neurologic symptoms	Imaging		Coinfections examined			Pre-ART viral loads (copies/ml)		
			Notes	Relevant citation	Negative	Positive	Relevant citation	Plasma	CSF	Relevant citation
San Francisco, CA, USA	355	Unsteady gait (myelopathy), cognitive impairment	Not done	(13)	[A]	HHV6/6B[B]	(42)			
	978	Aphasia, confusion & memory loss, personality change,	Axial T2-weighted images show patchy central peripheral white matter signal abnormality	(88)	[A]		(42)			
	1024	Cognitive impairment, gait instability, ataxia, impulsiveness	FLAIR images demonstrate patchy peripheral white matter abnormality	(88)	[A]		(42)			
	1078	Cognitive impairment								
	818	Cognitive impairment, personality change			[A]		(42)			
	351	Cognitive impairment, gait instability, leg spasticity			[A]		(42)			
	668	Cognitive impairment								
Pune, India	3164	Headache, gait instability, tremors, cognitive impairment			HBV[C], HCV[C], Syphilis[C], TB[C], cryptococcus[C], [A]	EBV[B], HHV7[B]	(42)			
	3163	Gait instability, tremors			HBV[C], HCV[C], Syphilis[C], TB[C], cryptococcus[C], [A]		(42)			
	3219	Gait instability, tremors, cognitive impairment			HBV[C], HCV[C], Syphilis[C], TB[C], cryptococcus[C], [A]	EBV[B]	(42)	687,135		
	3245	Headache, gait instability, tremors			HBV[C], HCV[C], Syphilis[C], TB[C], cryptococcus[C], [A]	EBV[B]	(42)			
	3309	Headache, gait instability, tremors, cognitive impairment, seizures			HBV[C], HCV[C], Syphilis[C], TB[C], cryptococcus[C], [A]	CMV[B], EBV[B]	(42)			
	3328	Gait instability, tremors, cognitive impairment, incontinence			HBV[C], HCV[C], Syphilis[C], TB[C], cryptococcus[C], [A]	EBV[B]	(42)	77,494		
	3452	Gait instability, tremors, cognitive impairment			HBV[C], HCV[C], Syphilis[C], TB[C], cryptococcus[C]					
	3313	Gait instability, tremors, cognitive impairment			HBV[C], HCV[C], Syphilis[C], TB[C], cryptococcus[C], [A]		(42)	124,509		
	3366	Gait instability, cognitive impairment			HBV[C], HCV[C], Syphilis[C], TB[C], cryptococcus[C]			42,993		
	3320	Headache, gait instability			HBV[C], HCV[C], Syphilis[C], TB[C], cryptococcus[C]					
	1481	Headache, gait instability, tremors			HBV[C], HCV[C], Syphilis[C], TB[C], cryptococcus[C]					
	3201	Gait instability, tremors, cognitive impairment, paralysis of limb, incontinence, seizures			HBV[C], HCV[C], Syphilis[C], TB[C], cryptococcus[C], [A]		(42)			
Milan Italy	4947	Memory loss	Normal MRI		[A]		(42)	21,015		
	7157	Headache, somnolence, dizziness	Oedema and T2 hyperintensity of the caudate, putamen, and lenticular nuclei and internal capsula, periventricular hyperintensity. No contrast enhancement.		[A]	EBV[B]	(42)			
	9544	Tremor, ataxia, progressive cognitive decline, memory loss	Leukoencephalopathy, atrophy		[A]		(42)			
Torino, Italy	Cal4	Dizziness, severe gait abnormalities, tremors	Abnormalities at the time of escape that improved over 9 months following ART optimization	(89)	CMV[C], JCV[C], HSV-1/2[C]	EBV[C]	(89)	557,351	2,545	(89)
Gothenburg, Sweden	9804	Severe HIV encephalitis			[A]		(42)	150,000		
	5168	Cognitive decline	Slight deformity of frontal ventricular horns, stable compared with previous examinations. Otherwise normal.	(13)	[A]	EBV[B]	(42)	4,120		

[A]Sample tested with global pathogen detection IDseq

[B]Pathogen identified with global pathogen detection IDseq method

[C]Pathogen tested for and/or identified by testing at the local clinical site

Information in this table with no reference listed was provided by the clinic that collected the sample

Supplemental Table 2. Comparator cohorts

Cohort	N	Country of origin	Sampling year range	On ART?	Neurosymptomatic?	**Plasma HIV-1 RNA (cp/ml)** median (IQR)	**CSF HIV-1 RNA (cp/ml)** median (IQR)	**Blood CD4 (cells/µl)** median (IQR)	**CSF WBC (cells/µl)** median (IQR)
NSE	25	USA, India, Italy, Sweden	2000-2019	Yes	Yes	86 (39-220)	1700 (726-4,700)	427 (380-660)	14 (12-44)
Untreated, chronic infection	7	USA	2012-2013	No	No	123,213 (43,883-167,727)	12,434 (4,487-35,606)	279 (215-368)	7 (3-14)
Untreated, primary infection	7	USA	2003-2009	No	No	367,728 (167,000-416,500)	15,200 (4,429-28,706)	705 (445-729)	7 (4-19)
ASE	2	USA	2013	Yes	No	39 (39-39)	780 (523-1038)	205 (203-208)	46 (23-66)
ART-suppressed	49	USA, Sweden	2006-2019	Yes	No	39 (39-39)	39 (39-39)	600 (440-784)	1 (0-3)

Supplemental Table 3. ART optimization and follow up visit information

	Initial NSE					ART Optimization			NSE Follow up					
Subject ID	Collection Date	NSE Plasma HIV RNA (cp/ml)	NSE CSF HIV RNA (cp/ml)	NSE Blood CD4 (cells/µl)	NSE CSF WBC (cell/µl)	Date of ART optimization	Date of follow up	Days between ART optimization and follow up	Follow up Plasma HIV RNA (cp/ml)	Follow up CSF HIV RNA (cp/ml)	Follow up Blood CD4 (cells/µl)	Follow up CSF WBC (cells/µl)	Did symptoms improve?	Notes
355	3/10/00	378	5467	262	47	3/12/00	4/7/00	26	<40	205	372	39	Yes	
978	3/18/13	<40	274	545	68	4/9/13	5/17/13	38	<40	<40	530	14	Yes	
818	4/26/10	57	1493	438	22	7/1/11	2/6/12	220	<40	<40	501	9	Yes	
9544	1/20/15	86	853	733	24	1/23/15	6/30/15	158	<40	<40	534	1	Yes	
Cal4	2/11/16	90	7566	784	44	2/16/16	5/27/16	101	<40	<40	N/A	N/A	Yes	
9804	10/16/14	<40	5182	1310	44	11/22/14	1/20/15	59	<40	<40	N/A	N/A	Yes	
3163	11/12/17	390	2100	695	5	12/11/17	7/10/18	211	<40	<40	NA	N/A	Yes	
3219	12/29/17	290	1700	322	6	12/29/17	4/4/19	461	320	5000	266	N/A	Yes	
3201	1/1/18	<20	4700	337	6	1/3/18	6/3/18	151	<40	<40	607	N/A	Yes	
3309	4/7/18	470	3100	396	31	7/10/18	6/4/19	329	<40	<40	887	N/A	Yes	
3313	11/7/18	140	7100	313	13	7/19/18	10/13/18	86	<40	<40	389	N/A	Yes	
3366	8/31/18	100	330	656	12	8/31/18	12/20/18	111	<40	<40	994	N/A	Yes	
3320	6/12/18	120	6800	267	N/A	12/6/18	8/3/19	240	<40	<40	310	N/A	Yes	
3452	12/29/18	27	910	574	6	1/1/19	9/23/19	265	<40	<40	938	N/A	Yes	
1024	4/23/14	48	3403	615	57	4/24/14	N/A	N/A	N/A	N/A	N/A	N/A	Yes	No CSF at follow up
4947	12/20/13	92	912	797	52	2/28/14	N/A	N/A	N/A	N/A	N/A	N/A	Yes	No CSF at follow up
5168	5/22/08	118	3230	660	9	3/30/10	4/29/10	30	<40	N/A	650	N/A	Yes	No CSF at follow up
3164	11/21/17	220	2700	194	12	N/A	N/A	N/A	470	NA	255	N/A	Yes	No CSF at follow up
3245	4/20/18	39	290	578	17	N/A	N/A	N/A	<40	NA	NA	N/A	Yes	No CSF at follow up
3328	3/8/18	290	6900	412	15	N/A	N/A	N/A	<40	NA	NA	N/A	Yes	No CSF at follow up
1481	2/25/19	62	620	427	12	N/A	N/A	N/A	<40	NA	421	N/A	Yes	No CSF at follow up

Supplemental Table 4. Mann-Whitney p values from Figure 5B

Biomarker	p values		
	**Lower diversity vs control**	**Higher diversity vs control**	**Lower diversity vs higher diversity**
CD27	< 0.0001	< 0.0001	0.1903
MXB1	< 0.0001	< 0.0001	0.4363
CD79b	< 0.0001	< 0.0001	0.2973
IFNg	< 0.0001	< 0.0001	0.3865
MMP9	< 0.0001	< 0.0001	0.4363
CD48	< 0.0001	< 0.0001	0.1615
TNFS14	< 0.0001	< 0.0001	0.8633
LTA	< 0.0001	< 0.0001	0.2973
FASLG	< 0.0001	< 0.0001	0.1615
CHI3L1	< 0.0001	< 0.0001	0.0939

Supplemental Table 5. Primer sequences

Primer	Sequence	HXB2 numbers for gene specific primer regions
SGA1 Forward	GGGTTTATTACAGGGACAGCAGAG	4900-4923
SGA1 Reverse	GCACTCAAGGCAAGCTTTATTGAGGCTTA	9632-9604
SGA2 Forward	CACCGGCTTAGGCATCTCCTATACCAGGAAGAA	5954-5982
SGA2 Reverse	CTGCCAATCAGGGAAGTAGCCTTGTGT	9171-9145
MiSeq Subtype B V3 cDNA[A]	GTGACTGGAGTTCAGACGTGTGCTCTTCCGATCTNNNNNNNNNNNCAGTCCATTTTGCTYTAYTRABVTTACAATRTGC	7238-7209
MiSeq Subtype C V3 cDNA[A]	GTGACTGGAGTTCAGACGTGTGCTCTTCCGATCTNNNNNNNNNGCTATGTGTTGTAATTTCTAGGTCCCCT	7322-7248
MiSeq PR cDNA[A]	GTGACTGGAGTTCAGACGTGTGCTCTTCCGATCTNNNNNNNNNCAGTTTAACTTTTGGGCCATCCATTCC	2614-2592
MiSeq IN cDNA[A]	GTGACTGGAGTTCAGACGTGTGCTCTTCCGATCTNNNNNNNNNNNATCGAATACTGCCATTTGTACTGC	4771-4752
MiSeq RT cDNA[A]	GTGACTGGAGTTCAGACGTGTGCTCTTCCGATCTNNNNNNNNNNNCAGTCACTATAGGCTGTACTGTCCATTTATC	3284-3258
MiSeq V3 PCR1	GCCTCCCTCGCGCCATCAGAGATGTGTATAAGAGACAGNNNNTTATGGGATCAAAGCCTAAAGCCATGTGTA	6555-6584
MiSeq PR PCR1	GCCTCCCTCGCGCCATCAGAGATGTGTATAAGAGACAGNNNNTCAGAGCAGACCAGAGCCAACAGCCCCA	2136-2163
MiSeq IN PCR1	GCCTCCCTCGCGCCATCAGAGATGTGTATAAGAGACAGNNNNAAAAGGAGAAGCCATGCATG	4364-4383
MiSeq RT PCR1	GCCTCCCTCGCGCCATCAGAGATGTGTATAAGAGACAGNNNNGGCCATTGACAGAAGAAAAAATAAAAGC	2620-2647
MiSeq PCR1 reverse primer	GTGACTGGAGTTCAGACGTGTGCTC	N/A
MiSeq PCR2 primer 1	AATGATACGGCGACCACCGAGATCTACACGCCTCCCTCGCGCCATCAGAGATGTG	N/A
MiSeq PCR2 primer 2[B]	CAAGCAGAAGACGGCATACGAGATNNNNNNGTGACTGGAGTTCAGACGTGTGCTC	N/A
Sequencing primer for MiSeq runs	GCCTCCCTCGCGCCATCAGAGATGTGTATAAGAGACAG	N/A

[A]Ns in the cDNA primer sequences are random primers

[B]In the index primer the 6-N is not a random primer but a pre-designed index sequence. We use a set of 24 indices

Supplemental Table 6. Sizes of amplicons sequenced

Sequencing method	Region	HXB2 numbers for sequenced regions
SGA/Sanger	env	6325-8794
MiSeq	V3	6585-7208[A]
MiSeq	PR	2164-2593
MiSeq	IN	4384-7517
MiSeq	RT	2648-2914 and 3001-3257[B]

[A]A small segment of C2 is missing
[B]These two sequence fragments were joined together

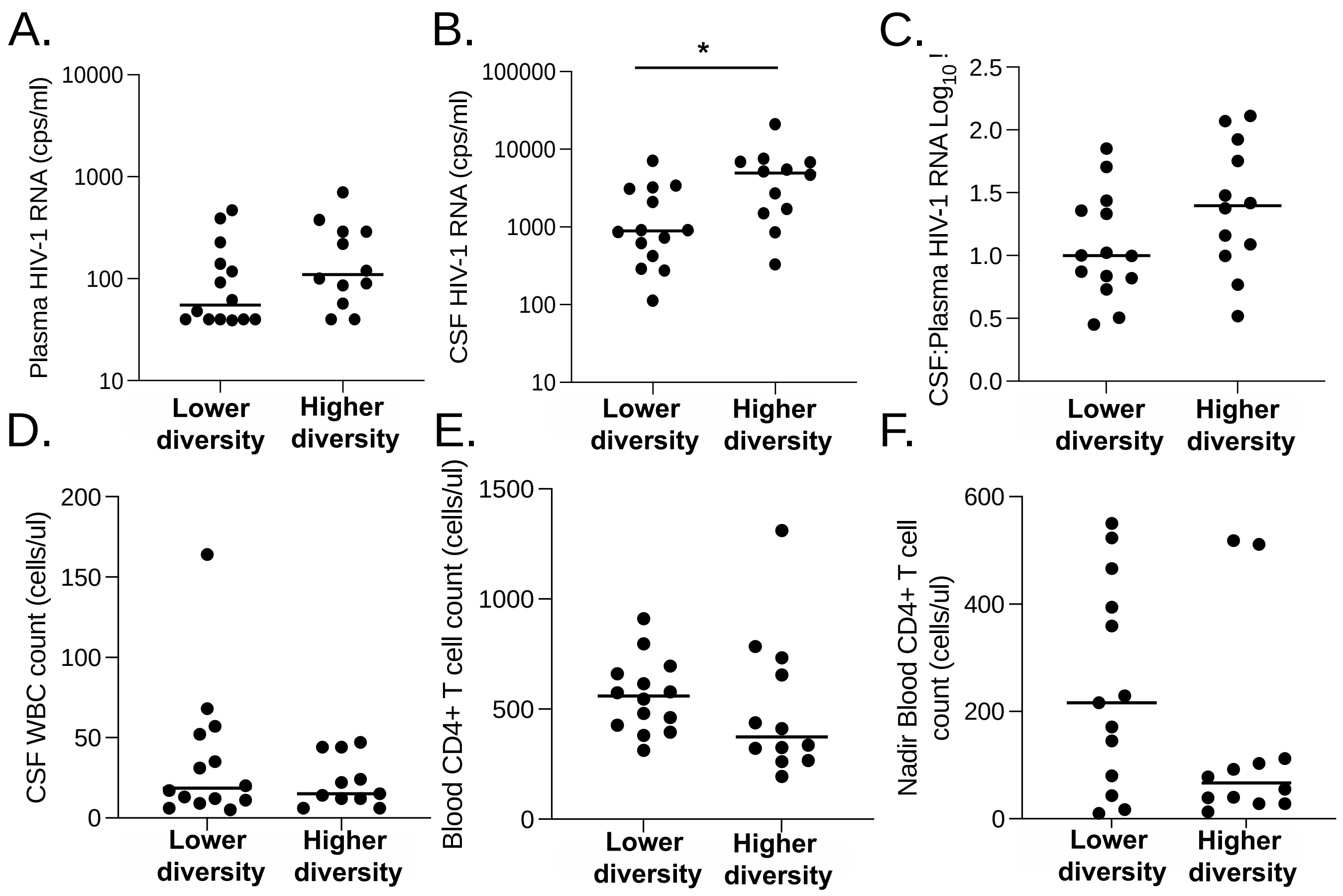

Supplemental Figure 1. CSF HIV-1 RNA levels are higher in NSE participants with more diverse NSE viral populations.

Comparisons were done for plasma HIV-1 RNA (**A**), CSF HIV-1 RNA (**B**), CSF:plasma HIV-1 RNA $\log_{10}$ Δ (**C**), CSF WBC count (**D**), blood CD4+ T cell count (**E**), and nadir blood CD4+ T cell count (**F**) in the NSE cohort (N = 25). Mann-Whitney tests were performed for all comparisons and found that CSF HIV-1 RNA levels were greater in participants with higher diversity NSE populations (mean PWD above 0.004) than in participants with lower diversity (mean PWD below 0.004) NSE populations (p = 0.03). All p values were corrected for multiple comparisons. Median values are shown with horizontal bars.

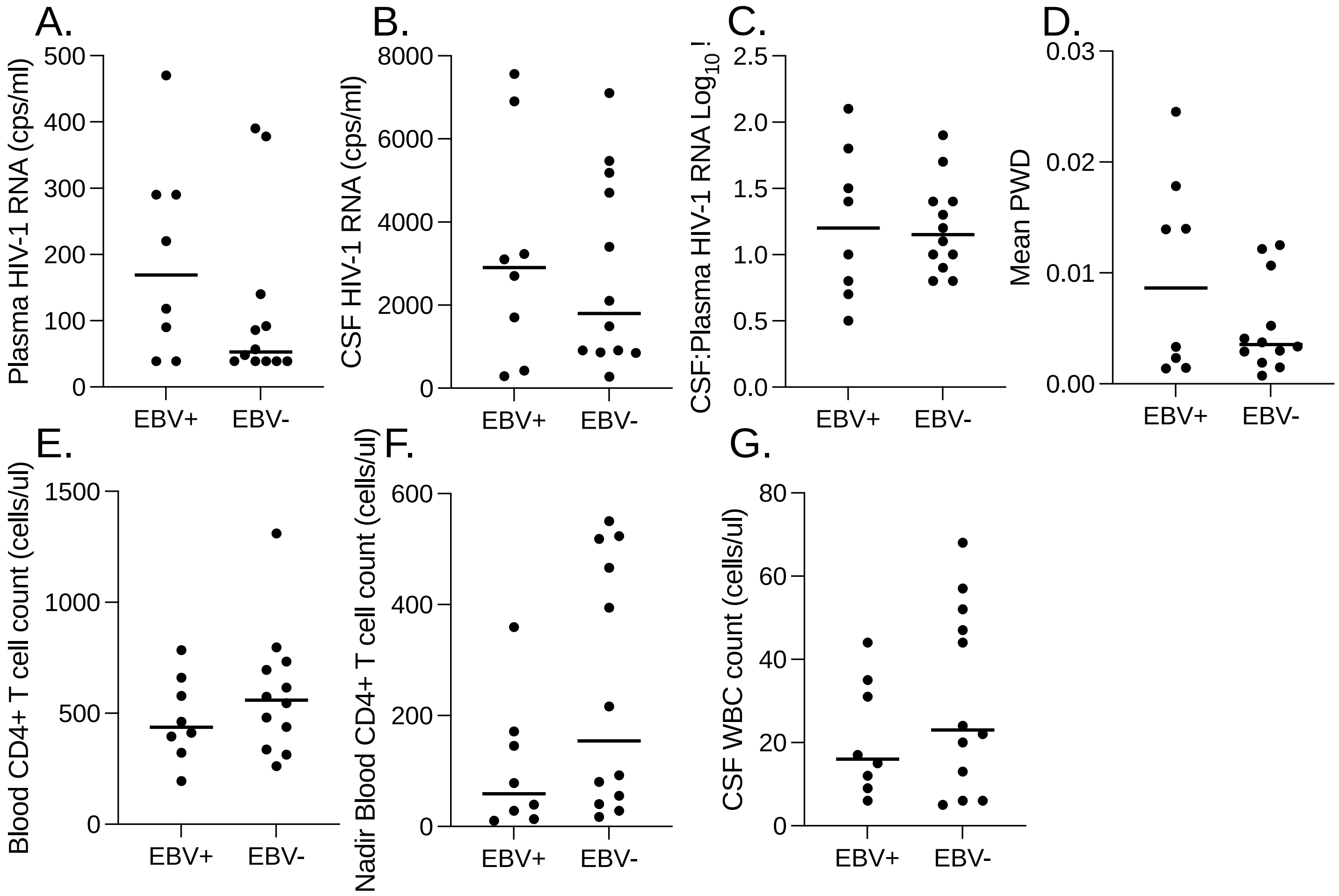

Supplemental Figure 2. NSE participants do not differ in HIV-1 RNA levels, cell counts, or mean PWD based on the presence of EBV in the CSF.

A subset of NSE participants were tested for CSF coinfections. The most common pathogen detected was EBV (found in 9/20 participants). We compared plasma HIV-1 RNA (**A**), CSF HIV-1 RNA (**B**), CSF:plasma HIV-1 RNA $\log_{10} \Delta$ (**C**), mean PWD (**D**), blood CD4+ T cell count (**E**), nadir blood CD4+ T cell count (**F**), and CSF WBC count (**G**) between EBV+ (N = 8) and EBV- (N = 12) participants. Mann Whitney tests found no significant differences in any of these comparisons. All p values were corrected for multiple comparisons. Median values are shown with horizontal bars.

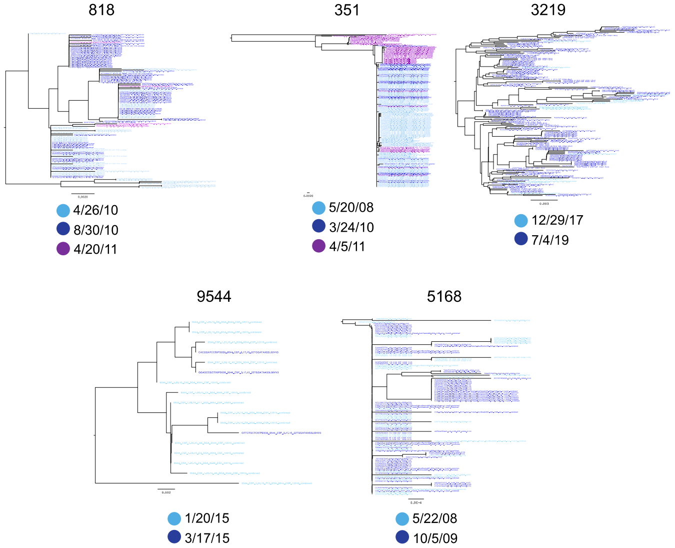

Supplemental Figure 3. Partial *env* sequences from participants with multiple NSE time points. Neighbor-joining phylogenetic trees for participants with multiple NSE timepoints (timepoint 1, light blue; timepoint 2, dark blue; timepoint 3 (if applicable), purple). Sample collection dates are shown. Evidence of ongoing replication and evolution can be seen in participant 351 with the mostly purple lineage at the top of the tree that has diverged from the light and dark blue sequences in the main part of the tree.

9

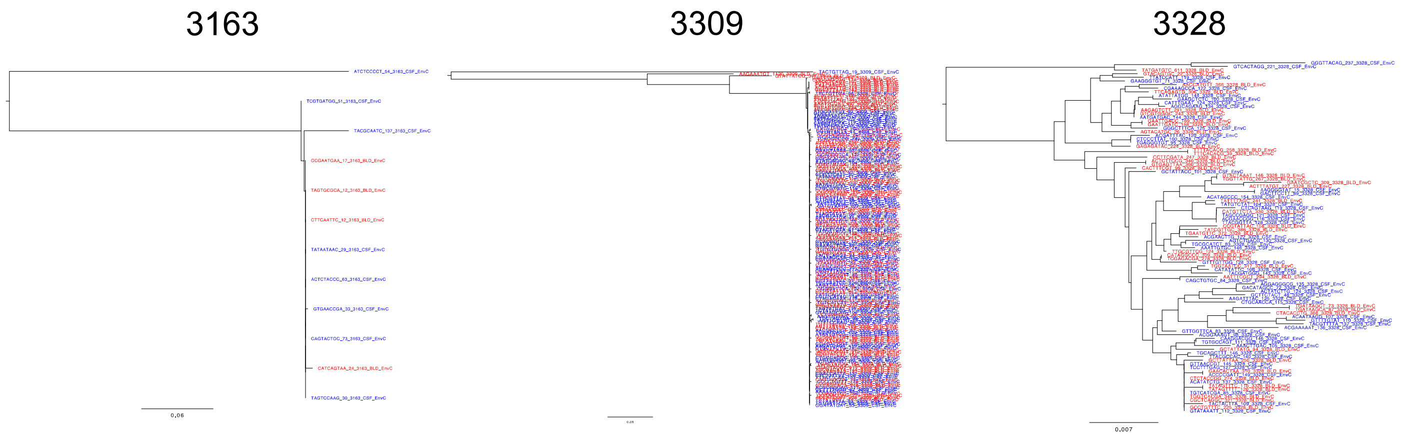

Compartmentalization tests for pictured trees
Slatkin Maddison p = 0.354
Structured Slatkin Maddison p = 0.999

Compartmentalization tests for trees with equal numbers of sequences from each compartment
Slatkin Maddison p = 0.757
Structured Slatkin Maddison p = 0.999

Compartmentalization tests for pictured trees
Slatkin Maddison p = 0.001
Structured Slatkin Maddison p = 0.318

Compartmentalization tests for trees with equal numbers of sequences from each compartment
Slatkin Maddison p < 0.001
Structured Slatkin Maddison p = 0.181

Compartmentalization tests for pictured trees
Slatkin Maddison p = 0.019
Structured Slatkin Maddison p = 0.736

Compartmentalization tests for trees with equal numbers of sequences from each compartment
Slatkin Maddison p = 0.064
Structured Slatkin Maddison p = 0.828

Supplemental Figure 4. Env sequences from the blood plasma are equilibrated with env sequences from the CSF during NSE.

Neighbor-joining phylogenetic trees from three representative participants for which we were able to generate blood (red) and CSF (blue) sequences at the time of escape. The trees pictured, as well as trees with equal numbers of blood and CSF sequences (to avoid false positives), were analyzed for compartmentalization (classical Slatkin Maddison and Structured Slatkin Maddison). In all participants, blood and CSF sequences were equilibrated.

References not in main text

88. Narvid J, et al. Brain MRI Features of CSF Human Immunodeficiency Virus Escape. *J Neuroimaging.* 2018;28(6):601-7.

89. Trunfio M, et al. Symptomatic cerebrospinal fluid HIV-1 escape with no resistance-associated mutations following low-level plasma viremia. *J Neurovirol.* 2018;24(1):132-6.
